# Segregation of D1 and D2 dopamine receptors in the striatal direct and indirect pathways: An historical perspective

**DOI:** 10.3389/fnsyn.2022.1002960

**Published:** 2023-01-19

**Authors:** Charles R. Gerfen

**Affiliations:** Section on Neuroanatomy, National Institute of Mental Health (NIMH), Bethesda, MD, United States

**Keywords:** striatum, basal ganglia, dopamine, Parkinson’s disease, motor function

## Abstract

The direct and indirect striatal pathways form a cornerstone of the circuits of the basal ganglia. Dopamine has opponent affects on the function of these pathways due to the segregation of the D1- and D2-dopamine receptors in the spiny projection neurons giving rise to the direct and indirect pathways. An historical perspective is provided on the discovery of dopamine receptor segregation leading to models of how the direct and indirect affect motor behavior.

## Introduction

Prevailing models of basal ganglia function are based on two main pathways originating from separate populations of the spiny projection neurons (SPNs) in the striatum that either directly or indirectly connect to output circuits that affect motor behavior. These direct and indirect striatal pathways were proposed to differentially promote and suppress actions in hyperkinetic and hypokinetic clinical disorders (Albin et al., 1989). The discovery that the D1 and D2 dopamine receptors are respectively expressed in direct and indirect spiny projection neurons (dSPNs and iSPNS) that give rise to these pathways provided the mechanism for the opponent effects of dopamine on their function (Gerfen et al., 1990). In Parkinson’s disease dopamine depletion in the striatum produces physiologic alterations in both pathways responsible for L-DOPA induced dyskinesias (LIDs) (Fieblinger et al., 2014). The insight that the normal performance of behavior is dependent on activity in both pathways led to the proposal that the indirect pathway suppresses alternative actions that would compete with selected actions encoded by activity in the direct pathway (Mink, 1996). Development of molecular genetic research tools confirmed this model by demonstrating concurrent opponent activity in dSPNs and iSPNs during the performance of normal actions (Cui et al., 2013; Jin et al., 2014; Tecuapetla et al., 2016). Recent work has revealed the contribution of subtypes of neurons in the external segment of the globus pallidus (GPe), which themselves exert opponent effects on the performance of motor behavior (Pamukcu et al., 2020; Aristieta et al., 2021; Ketzef and Silberberg, 2021; Cui et al., 2021a,b). Current models demonstrate that activity at multiple levels in the direct and indirect pathways provide opponent effects that underlie the functional role of the basal ganglia during the complex performance of motor behavior.

From a historical perspective research on the basal ganglia pioneered neuroanatomical approaches for understanding the organization of brain circuits underlying brain disorders. Seminal work by Arvid Carlsson and Oleh Hornykiewicz in the late 1950s-early 1960s established that Parkinson’s disease results from the degeneration of dopamine systems in the basal ganglia. This led to the development of a precursor of dopamine, L-DOPA as an effective treatment that reversed the bradykinesia symptomatic of the disease (Hornykiewicz, 1966).

Carlsson et al. (1962) used the discovery by Falck and Hillarp that catecholamines in brain tissue, including dopamine, exposed to formaldehyde condensed into a fluorescent molecule, to visualize catecholamine containing neurons and processes in brain sections. This technique was used by several Swedish scientists, including Fuxe, Dahlstrom, Bjorklund among others to map the distribution of dopaminergic and noradrenergic brain systems (Dahlström and Fuxe, 1964; Fuxe, 1965). Included in these studies was the identification of dopaminergic neurons in the substantia nigra that project axons to the striatum, the nigrostriatal dopamine system. Ungerstedt (1968) used the neurotoxin 6-OHDA, an analog of dopamine that is selectively taken up by dopaminergic and other catecholaminergic neurons, to lesion the nigrostriatal dopamine system resulting in bradykinetic behavior similar to the clinical deficits of Parkinson’s disease. These studies established the experimental strategy of functional neuroanatomy to map neurochemically defined brain circuits and the experimental strategy to selectively target and alter the function of these circuits to determine their role in behavior.

**FIGURE 1:**
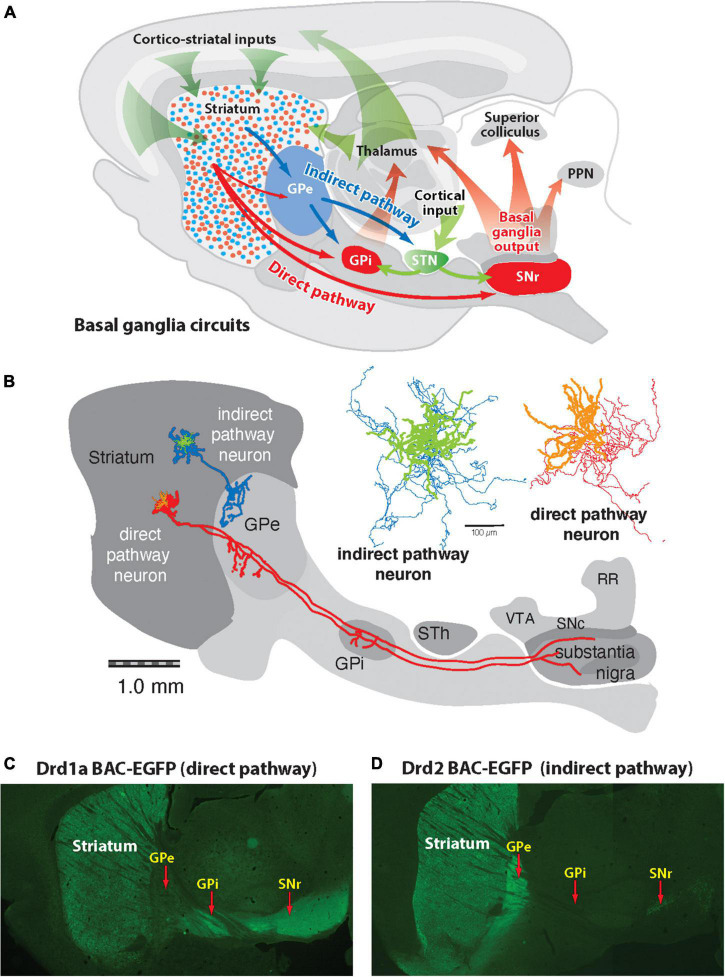
Diagram of basic basal ganglia circuits. **(A)** The striatum receives excitatory corticostriatal and thalamic inputs. Outputs of the basal ganglia arise from the internal segment of the globus pallidus (GPi) and substantia nigra pars reticulata (SNr), which are directed to the thalamus, superior colliculus, and pedunculopontine nucleus (PPN). The direct pathway originates from Drd1a-expressing SPNs that project to the GPi and SNr output nuclei. The indirect pathway originates from Drd2-expressing SPNs that project only to the external segment of the globus pallidus (GPe), which together with the subthalamic nucleus (STN) contain transynaptic circuits connecting to the basal output nuclei. **(B)** Tracings of individual indirect and direct striatal projection neurons drawn in place on a sagittal brain diagram (Kawaguchi et al., 1989). The indirect striatal pathway neuron (green) has axon collaterals (blue) that spreads locally within the striatum and one that projects into the GPe, where it arborizes, and does not extend beyond this nucleus. The direct striatal pathway neuron (orange) has a local collateral within the striatum and projection axons (red) that extend some collaterals into the globus pallidus (GPe), and others to the globus pallidus internal segment (GPi) and substantia nigra. **(C)** Fluorescent imaging of a brain section from a mouse expressing enhanced green fluorescent protein (eGFP) under regulation of the Drd1a promoter shows Drd1a-expressing SPNs in the striatum that project axons through the GPe, which terminate in the GPi and GPe. **(D)** Fluorescent imaging of a Drd2-eGFP mouse shows that labeled SPNs provide axonal projections that terminate in the GPe but do not extend to the GPi or SNr.

The basic structural organization of the basal ganglia was described by Nauta and Mehler (1966) in the first paper of the journal Brain Research. Using silver impregnation of degenerating axons, they identified separate pathways from the striatum to the external and internal segments of the globus pallidus (GPe and GPi) as well as projections from the GPi and SNr to the thalamus and midbrain motor areas. Advances in techniques in the 1980s greatly expanded the ability to map neuroanatomical circuits with greater detail. These included immunohistochemical localization of neurochemicals, receptor binding methods, *in situ* hybridization histochemistry (ISHH) to localize genes expressed in neurons, retrograde and anterograde axonal tracing methods and intracellular labeling of the axonal projections of individual striatal neurons. These techniques were used to describe the direct and indirect striatal pathways that form the conceptual backbone of the functional organization of the basal ganglia (Gerfen and Wilson, 1996; Smith et al., 1998). The striatum is the major input nucleus receiving excitatory inputs from most areas of the cerebral cortex and the intralaminar thalamus, as well as dopaminergic inputs from the substantia nigra pars compacta (Figure 1A). The main output of the basal ganglia originate from GABAergic neurons in the internal segment of the globus pallidus (GPi) and substantia nigra pars reticulata (SNr). Their output is directed to thalamic nuclei and midbrain areas involved in motor function. The direct and indirect pathways refer to connections from striatal SPNs to the output nuclei of the basal ganglia. Axonal projections of direct pathway SPNs (dSPNs) provide inputs directly to the output nuclei, whereas indirect pathway SPNs (iSPNs) project only to the external segment of the globus pallidus (GPe) whose neurons provide indirect connections with the output nuclei of the basal ganglia, including connections through the subthalamic nucleus (STN). Among variations in the details of the connections from the cerebral cortex through the basal ganglia, are the patch/striosome and matrix compartmentation of striatal inputs and outputs (Gerfen, 1984, 1992; Jiménez-Castellanos and Graybiel, 1989) and regional differences related to different functions of the cortical areas providing inputs (Alexander et al., 1986). Nonetheless, the direct and indirect pathways are a core organizing feature underlying basal ganglia function.

Definitive determination that distinct striatal SPNs give rise to the direct (dSPN) and indirect (iSPN) output pathways was provided by a technique developed by Kitai et al. (1976), Chang et al. (1981), Bishop et al. (1982), Kawaguchi et al. (1989) to intracellularly fill neurons with a marker that labeled both the dendrites and axons of individual neurons (Figure 1B). Individual dSPNs displayed a local collateral axon plexus that spread in an area of 300 μm around the cell body and a projection axon that passed through the GPe where it extended a collateral and then coursed through the internal capsule to provide a terminal plexus in both the GPi and SNr. Individual iSPNs also displayed a local collateral around the cell body while a projection axon extended into the GPe where it ramified into a terminal plexus typically in two areas of the GPe but did not extend an axon beyond the GPe. Transgenic mice, in which the entire population of either striatal dSPNs or iSPNs are labeled by GFP or Cre recombinase expression under the D1- or D2-dopamine receptors (Gong et al., 2003; Gerfen et al., 2013) display the full pattern of these striatal projection pathways (Figures 1C, D). While iSPNs connect monosynaptically only with the circuits of the indirect pathway, dSPNs not only provide direct input to the output nuclei of the basal ganglia but also connect with the indirect pathway through the axon collateral to the GPe. The collateral of the dSPNs to the GPe has sometimes been overlooked as being a sparse connection and was often unlabeled with retrograde tracers injected into the GPe. However, functional significance of this bridging collateral between the direct and indirect pathways is indicated by the finding that the anatomical extent of the axon within the GPe appears to be dynamically altered inversely related to excitability of iSPNs (Cazorla et al., 2014).

That dSPNs and iSPNs could be distinguished based on their neurochemical phenotype was first demonstrated with immunohistochemical localization of the neuropeptides substance P and enkephalin (Beckstead and Kersey, 1985). That dopamine differentially affected dSPNs and iSPNs was demonstrated by increased expression of substance P in the SNr following dopamine agonist treatments, while D2 receptor antagonists increased expression of enkephalin in the GPe (Li et al., 1987). Development of *in situ* hybridization labeling of mRNA transcripts (ISHH) added a powerful technique that advanced characterization of striatal neurons giving rise to the direct and indirect pathways. Using this approach, ISHH localization of neurons expressing substance P or enkephalin mRNAs combined with fluorescent retrograde axonal tracers confirmed that dSPNs express substance P mRNA whereas iSPNs express enkephalin mRNA (Gerfen and Young, 1988). Following unilateral lesions of the nigrostriatal dopamine system with 6-OHDA levels of substance P mRNA in dSPNs was shown to decrease whereas levels of enkephalin mRNA in iSPNs was shown to increase (Young et al., 1986). This study demonstrated the opposing effects of dopamine on dSPNs and iSPNS.

Initial models of how the functional organization of basal ganglia circuits affect behavior focused on the direct pathway. Physiologic studies demonstrated that cortical input to the striatum provides excitatory activation of the GABAergic dSPNs, which in turn inhibit the GABAergic neurons in the SNr (Deniau and Chevalier, 1985). As the GABAergic neurons of the SNr are tonically active, activation of the striatonigral direct pathway disinhibits the targets of inhibitory input to the thalamus and superior colliculus (Chevalier et al., 1985). The behavioral effect of this disinhibition was demonstrated as the pauses in SNr activity were correlated with increased activity in the superior colliculus during saccadic eye movements (Hikosaka and Wurtz, 1983). These studies suggested that cortical inputs to the striatum activating the direct pathway are involved in the generation of motor behavior.

In a landmark paper Albin et al. (1989) synthesized findings of pharmacologic affects and pathologic changes in basal ganglia circuits to propose that hyperkinetic and hypokinetic clinical movement disorders result from imbalances in activity of the direct and indirect striatal output pathways. Hypokinetic disorders, such as the bradykinesia symptomatic of Parkinson’s disease, was suggested to result from decreased activity in the direct pathway relative to the indirect pathway while hyperkinetic disorders, such as chorea, ballism and tics were suggested to result from the opposite imbalance. Evidence of the involvement of the direct pathway in hypokinetic disorders came from studies demonstrating decreased markers in the direct striatonigral pathway in various animal models of Parkinson’s disease. That decreased function in the direct pathway would result in diminished motor function was consistent with the model that activation of the direct pathway to inhibit the GABAergic neurons in the SNr and GPi, would result in disinhibition of their targets in the thalamus and motor areas of the midbrain, including the superior colliculus. Thus, in the normal condition, increased activity in the direct striatonigral pathway produces pauses in the GABAergic inhibitory basal ganglia output, which had been shown to be correlated with production of movements such as saccadic eye movements (Hikosaka and Wurtz, 1983). In their model of the role of the indirect pathway in movement disorders Albin et al. (1989) incorporated the contribution of the STN. The indirect pathway is composed of the projections of the iSPNs to the GABAergic neurons in the external segment of the globus pallidus (GPe), which provide inputs to glutamatergic neurons in the STN, which provide excitatory input to the SN and GPi, the output nuclei of the basal ganglia. While there is considerably more complexity in the indirect pathway, in the model of hyperkinetic disorders, decreased activity in the iSPNs disinhibits GPe neurons resulting in decreased activity of STN neurons, to decrease the inhibitory output of the basal ganglia was proposed to underlie abnormal motor behaviors including chorea, ballism and dyskinesia. Increased activity through the indirect pathway would increase the inhibitory output of the basal ganglia to diminish motor activity.

Taken together these findings led to the proposal that in Parkinson’s disease the loss of striatal dopamine produces decreased activity in the direct striatonigral pathway and increased activity in the indirect pathway through the multisynaptic pathway through the GPe and STN to SNr. Imbalance in activity between these pathways, with activity in the indirect pathway predominating over the disinhibition of direct pathway activity required for the generation of movements was proposed to produce bradykinetic symptoms of Parkinson’s disease. This model predicted that disruption of the indirect pathway would reverse the Parkinsonian bradykinesia. Substantiation of this model was provided by the reversal of bradykinesia in a non-human primate model of Parkinson’s disease with lesions of the STN (Bergman et al., 1990). The model of imbalances in the activity of the direct and indirect pathway following the depletion of dopamine in the striatum in Parkinson’s disease led directly to effective therapeutic surgical treatments directed at the indirect pathway. DeLong pioneered the lesions of the GPi in Parkinsonian patients (DeLong, 1990), followed by deep brain stimulation of the STN (Benabid, 2003), both of which proved highly effective in reversing bradykinesia in Parkinsonian patients.

Models of the role of the direct and indirect pathways in motor disorders of the basal ganglia led directly to the development of effective new approaches for the treatment of Parkinson’s disease (DeLong, 1990) but did not provide the mechanism of how dopamine had opposing effects on these circuits. Dopamine acts on at least 2 distinct receptors, the D1 and D2 dopamine receptors, (Cools and Van Rossum, 1976; Kebabian and Calne, 1979; Stoof and Kebabian, 1981; Creese et al., 1983) which are G protein-coupled receptors (GPCRs). In the case of dopamine receptors, ligand binding to GPCRs results in conformational changes in the coupling to G proteins that regulate the activity of adenylate cyclase production of cAMP, which activates downstream protein kinases responsible for gene expression. The D1 dopamine receptor (Drd1) is coupled to stimulatory G proteins (Gs or Golf) while the D2 dopamine receptor (Drd2) is coupled to the inhibitory G protein (Gi). Thus, dopamine acting through these receptors either increase, through Drd1 coupled Gs/Golf, or inhibit, through Drd2 coupled Gi, signal transduction pathways generating cAMP that leads to the regulation of various genes and their products. Studies had demonstrated that the dopamine agonist apomorphine selectively increased substance P immunoreactivity in dSPNs while Drd2 antagonists selectively increased enkephalin immunoreactivity in iSPNs (Li et al., 1987). These findings raised the possibility that dopamine differentially regulated the direct and indirect pathways through Drd1 and Drd2 receptors. However, a number of studies suggested that Drd1 and Drd2 receptors were co-localized in striatal projection neurons based on synergistic effects on the physiologic responses of SPNs when the receptors were activated together (Carlson et al., 1987; Bertorello et al., 1990).

**FIGURE 2:**
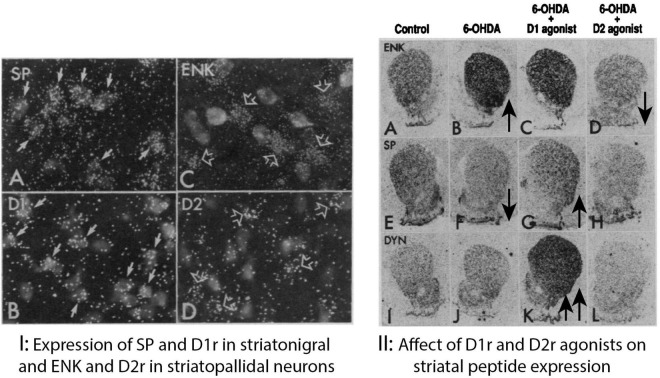
**(I)** Expression of SP and D1 in striatonigral and ENK and D2r in striatopallidal neurons. Striatal neurons retrogradely labeled with the fluorescent dye fluorogold after injection into the substantia nigra combined with darkfield illumination of silver grains produced by ISHH labeling with 35S labeled oligonucleotide probes for **(A)** substance P (SP), **(B)** the DI dopamine receptor (DI), **(C)** enkephalin (ENK), and **(D)** the D2 dopamine receptor (D2). Striatonigral neurons show ISHH labeling for both substance P [**(A)**, solid arrows] and the D1 dopamine receptor [**(B)**, solid arrows]. Striatal neurons that are unlabeled by fluorogold, and presumably project to the globus pallidus, show ISHH labeling for both enkephalin [**(C)**, open arrows] and the D2 dopamine receptor [**(D)**, open arrows]. **(II)** *In situ* hybridization in the striatum from brain sections apposed to autoradiographic film labeled with 35S-labeled oligonucleotide probes complementary to **(A–D)** enkephalin (ENK), **(E–H)** substance P (SP), and **(I–L)** dynorphin (DYN). Sections in the first two columns are from the same saline-treated animal showing ISHH labeling on the unlesioned control side **(A,E,I)** and lesioned 6-OHDA-injected side **(B,F,J)**. Sections in the third column are from the lesioned, 6-OHDA-injected side of an animal that received intermittent treatment with the D1 receptor selective agonist SKF38393 **(C,G,K)**. Sections in the fourth column are from the lesioned, 6-OHDA-injected lesioned side of animal that received continuous treatment with the D2 agonist quinpirole **(D,H,L)**. The increase in enkephalin ISHH labeling caused by 6-OHDA lesions **(B)** is not affected by D1 agonist treatment **(C)** but is reversed by continuous D2 agonist treatment **(D)**. The decrease in substance P ISHH labeling caused by 6-OHDA lesions **(F)** is reversed by intermittent D1 agonist treatment **(G)** but is unaffected by D2 agonist treatment **(H)**. Dynorphin ISHH labeling is not significantly altered by 6-OHDA lesions **(J)** but is elevated by D1 agonist treatment **(K)** but not affected by D2 agonist treatment **(L)** (Gerfen et al., 1990).

Demonstration that the Drd1 and Drd2 receptors are segregated respectively in the striatal neurons of the direct and indirect pathway provided a mechanism to explain how depletion of striatal dopamine would have opposite effects on these pathways (Gerfen et al., 1990). We had the idea that if Drd1 and Drd2 receptors were segregated in the direct and indirect pathways, then treatments with selective receptor agonists should selectively reverse the changes in neuropeptide expression levels in these pathways in the dopamine depleted striatum. Additionally, colleagues at NIH had cloned the mRNAs encoding the D1 and D2 dopamine receptors (Monsma et al., 1989, 1990), which enabled us to produce oligonucleotide probes to localize the receptors with ISHH (Gerfen et al., 1990). To determine which striatal neuron type expressed Drd1 and Drd2 receptors, the retrograde fluorescent tracer fluorogold was injected into the substantia nigra to label dSPNs. This resulted in labeling of approximately 43% of striatal neurons so that non-labeled neurons were presumed to be mostly striatal indirect SPNs. Radioactive S35 labeled oligonucleotide probes were used to label neurons with ISHH expressing either the Drd1 and Drd2 receptors, and neuropeptides expressed selectively by the iSPNs (enkephalin) or by dSPNs (Substance P and Dynorphin). To visualize neurons labeled with fluorogold and radioactive ISHH probes sections through the striatum were dipped in photographic emulsion and later developed, allowing visualization of silver grains over cells either labeled or not with the fluorescent tracer (Figure 2I). Results demonstrated that over 80% of cells labeled with either the Drd1, SP or DYN oligonucleotide probes were also labeled with fluorogold in striatonigral neurons. On the other hand, over 85% of cells labeled with either the Drd2 or ENK probes were not labeled with fluorogold. These results were supported by subsequent studies (Le Moine et al., 1990).

These results demonstrated that for the vast majority of striatal neurons the expression of Drd1 and Drd2 receptors are segregated respectively to dSPNs and iSPNs. However, a number of studies using single cell PCR, physiologic techniques or dopamine receptor immunoreactivity reported Drd1 and Drd2 receptor co-expression in a large percentage of striatal neurons (Surmeier et al., 1992, 1993; Ariano et al., 1993). Advances in ISHH techniques allowing the ability to visualize Drd1 and Drd2 receptor mRNAs with non-radioactive techniques in the same brain section (Young and Gerfen, unpublished), development of specific D1 and D2 dopamine receptor antibodies (Levey et al., 1993), the generation of transgenic mouse lines in which fluorescent markers are driven under the genetic control of D1 or D2 dopamine receptor expression (Gong et al., 2007; Gerfen et al., 2013) and single cell RNA expression profiling (Saunders et al., 2018) have provided a consensus that D1 and D2 receptors are segregated in all but less than 2% of striatal neurons (Gagnon et al., 2017). Although a small percentage of the total population of SPNs, those neurons co-expressing Drd1 and Drd2 dopamine receptors are reported to have significant unique effects on motor behavior (Bonnavion et al., 2022).

The functional significance of the segregation of dopamine receptors in SPNs was demonstrated by the effects that selective activation of these receptors had on functional measures (Figure 2II). We used the paradigm in which striatal levels of neuropeptides and dopamine receptor ISHH labeling was measured in animals with unilateral lesions of the nigrostriatal dopamine pathway with 6-OHDA that produced greater than 90% dopamine depletion in the striatum. Animals were then treated with saline or with either a Drd1 agonist (SKF38393, 12.5 mg/kg) or a Drd2 agonist (quinpirole, 1 mg/kg) for 21 days using daily intraperitoneal injections or continuous infusions with an osmotic minipump. In saline treated control animals, the 6-OHDA lesions produced a significant increase in ENK and Drd2 receptor mRNA and significant decreases in SP and Drd1 mRNA with little change in DYN mRNA. Continuous treatment with the Drd2 agonist reversed both the lesion induced increases in ENK and the Drd2 receptor while not affecting mRNA levels of SP, DYN or the Drd1 receptor. Daily intermittent treatments with the Drd2 agonist did not significantly affect any of the mRNA levels. Continuous treatment with the Drd1 agonist also did not significantly affect mRNA levels of any of the neuropeptides or receptors. Daily intermittent Drd1 receptor agonist treatments reversed the lesion induced decrease in both SP and the Drd1 receptor as well as a greater than 5-fold increase in DYN mRNA levels above control levels.

These changes in mRNA expression patterns were consistent with expected effects of the Drd1 and Drd2 receptors being expressed selectively in dSPNs and iSPNs. When occupied by dopamine, Drd1 receptors on dSPNs activate selected gene expression through the Gs signal transduction coupling, which is decreased in the absence of dopamine following nigrostriatal lesions. Treatment with selective Drd1 receptor agonists activates Gs signal transduction, reversing the dopamine depletion affects in the case of SP and D1 receptor expression and greatly increasing gene expression of other markers such as DYN. Opposite effects of dopamine on iSPNs are a consequence of their expression of the Drd2 receptor coupled through Gi to reduce signal transduction mediated selected gene expression. That removal of this inhibition following striatal dopamine depletion in Parkinson’s disease or animal models would increase function of the indirect pathway is supported by the finding that Drd2 agonist treatment reverses such increases. Together, neuroanatomical data and the effects of select D1- and D2-receptor agonists on gene expression provided evidence that dopamine has opponent functional effects on the dSPNs and iSPNs as a consequence of the segregation of the Drd1 and Drd2 receptors in these neurons (Gerfen et al., 1990).

## Altered function of dSPNs and iSPNs in Parkinson’s disease models

While some of the altered functions in dSPNs and iSPNs in the Parkinson’s disease animal model are reversed by subsequent selective dopamine receptor agonist treatment, other changes suggest that striatal dopamine depletion produces a change in the coupling of the Drd1 receptor to signal transduction systems. Following striatal dopamine depletion, Drd2 agonist treatment reversed the increase in Enk in iSPNs and Drd1 agonist treatment reversed the decrease in SP in dSPNs, but produced increased expression of Dyn above that in the dopamine intact striatum (Figure 2II). This suggested that Drd1-mediated regulation of expression of some genes is altered following dopamine denervation. Initial demonstration that Drd1 and Drd2 agonists have selective effects on gene regulation of neuropeptides was followed by studies demonstrating that immediate early genes (IEGs), such as c-fos, are also selectively induced in dSPNs and iSPNs. Induction of IEGs following pharmacologic treatments occur rapidly and thus provide a more direct measure of receptor mediated function that correlates with neuron activity (Paul et al., 1992; Robertson et al., 1992). In animals with striatal dopamine depletion, Drd1 agonist treatment induced c-fos in dSPNs in the lesioned striatum. Opposite effects on c-fos induction in iSPNs were produced with Drd2 agents. Antagonist treatment induced c-fos in iSPNs and not dSPNs, while agonists induced c-fos in the GPe, consistent with the inhibitory effect of dopamine on iSPNs. Induction of c-fos in dSPNs in the dopamine depleted striatum following Drd1 agonists is accompanied by contraversive rotation (Paul et al., 1992). In the initial unilateral 6-OHDA lesion rodent model of Parkinson’s disease dopamine agonist treatment produced a contraversive rotational behavior that was attributed to a supersensitive response produced by increased dopamine receptor expression to compensate for dopamine depletion (Ungerstedt, 1971a,b). However, following dopamine depletion in the striatum Drd1 expression decreases, which was reversed following agonist treatment (Gerfen et al., 1990). Induction of c-fos and other IEGs occurs in the dopamine depleted striatum after the first treatment with a dopamine agonist, when Drd1 levels are reduced, indicating that supersensitive induction was not due to increased expression of the Drd1 receptor (Gerfen et al., 2002).

**FIGURE 3:**
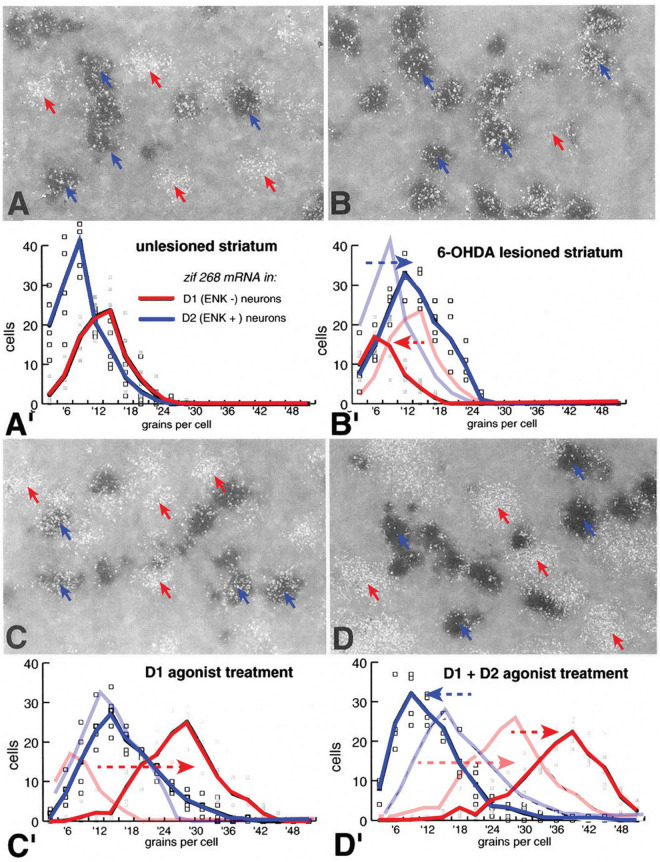
Expression of the IEG, zif268 (egr2) in D1r- and D2r-neurons in the control dopamine intact striatum **(A)**, in the dopamine-depleted 6-OHDA lesioned striatum **(B)**, in the lesioned striatum of animals treated with a D1r-selective agonist **(C)** and in the lesioned striatum of animals treated with both a D1r- and D2r-selective agonists **(D)**. Expression of zif268 is determined by the number of silver grains (white) generated with 35S-oligonucleotide labeling of zif268 mRNA concentrated over striatal neurons labeled with alkaline-phosphatase labeled ribonucleotide probes for ENK (blue arrows) or over putative D1r expressing neurons that do not express ENK (red arrows). Histogram distribution of the average number of zif268_ISHH generated silver grains per ENK+ (D2) and ENK– (D1) cells from five animals are plotted for each condition. In the unlesioned striatum there is no significant difference in zif268 levels between D1 and D2 cells **(A’)**. In the 6-OHDA lesioned striatum there is a significant increase in D2 cells and decrease in D1 cells **(B’)** compared to levels in the dopamine intact striatum (distributions from panel **A’** are shaded in panel **B’**). In animals with 6-OHDA lesions to deplete striatal dopamine that were treated with a D1r selective agonist (SKF38393, 1.0 mg/kg i.p.), there is no significant change in zif268 levels in D2 cells but a significant increase in D1 cells **(C’)** compared to levels in the dopamine depleted striatum without agonist treatment (distributions from panel **B’** are shown shaded in panel **C’**). Treatment with combined D1r-agonist (SKF38393, 1.0 mg/kg i.p.) and D2r-agonist (quinpirole, 1.0 mg/kg) resulted in a reduction in zif268 levels in D2-cells and a significant increase in D1-cells compared to levels compared with levels in the lesioned striatum of animals treated with the D1r-agoinist alone (distribution levels from panel **C’** are shaded in panel **D’**). These data demonstrate that D1r- and D2r-selective agonists have opposite acute effects on dSPN and iSPNs (Gerfen et al., 1995).

Studies had demonstrated that combined treatment with both D1 and D2 receptor agonists resulted in a synergistic response to increase c-fos induction in dSPNs in the dopamine depleted striatum (Paul et al., 1992). The question was how such synergy occurs as D1 and D2 receptor agonists produce increased and decreased activity respectively in dSPNs and iSPNs due to the segregation of the receptors. To analyze this synergy, we used the IEG zif268 (egr1), which in the basal condition is expressed at moderate levels to provide a measure of both increased and decreased responses in dSPNs and iSPNs (Gerfen et al., 1995). The experimental paradigm included using radioactively labeled zif268 antisense mRNA probes and the non-radioactive Enk antisense mRNA probe to label iSPNs in animals with unilateral lesions of the nigrostriatal dopamine pathway treated with different doses of a selective Drd1 agonist alone or in combination with a selective Drd2 agonist. Sections through the striatum were processed to label iSPNs with the histologic chromagen digoxigenin and zif268 mRNA levels by processing sections dipped in photographic emulsion producing silver grains that were used to quantify the level of zif268 expression at the single cell level in Enk positive iSPNs and Enk negative neurons presumed to be dSPNs. While many c-fos studies measured the number of labeled cells in response to various treatments, the method used provided the level of IEG labeling on a per cell basis. Results demonstrated that in the dopamine intact striatum there were basal levels of zif268 in both dSPNs and iSPNs (Figure 3A), while in the dopamine depleted striatum compared to the control zif268 levels decreased in dSPNs and increased in iSPNs (Figure 3B), parallel those of changes in peptide gene expression. In the lesioned striatum of animals treated with a selective Drd1 agonist zif268 levels in dSPNs increased significantly compared to levels in animals not receiving treatment, while in iSPNs there was no change (Figure 3C). In the lesioned striatum of animals treated with combined selective Drd1 and Drd2 agonists, there was a significant decrease in zif268 levels in iSPNs and a further increase in zif268 levels in dSPNs compared to those treated with D1-agonist alone (Figure 3D). This study provided further evidence that function of dSPNs and iSPNs are oppositely affected by their respective selective expression of Drd1 and Drd2 receptors. Moreover, the synergistic effects of combined Drd1 and Drd2 agonist treatment could result from direct interaction between iSPNs and dSPNs suggested by the further increase in dSPN response to Drd1 agonist coincident with decreased iSPN function. Connections between SPNs through their local collaterals has long been proposed to sculpt patterns of motor behavior (Groves, 1983). Physiologic studies demonstrated GABAergic transmission that through local collaterals SPNs may directly affect depolarization of neighboring SPNs to regulate spiking activity (Czubayko and Plenz, 2002). There are collateral connections between dSPN pairs and between iSPN pairs, but there are significantly more connections from iSPNs onto dSPNs with iSPNs providing much stronger affect (Taverna et al., 2008). In Parkinson’s disease models the physiologic connections between SPNs was reduced (Taverna et al., 2008) differentially affecting patterns of activity of dSPNs and iSPNs (López-Huerta et al., 2013; Parker et al., 2018).

**FIGURE 4:**
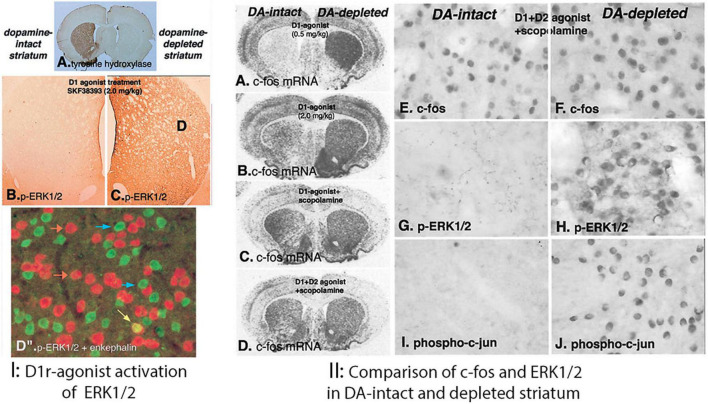
**(I)** D1 dopamine receptor-mediated phosphorylation of ERK1/2 (p-ERK1/2) in the dopamine-depleted striatum. Unilateral lesion of the nigrostriatal dopamine systems is demonstrated by the loss of tyrosine hydroxylase immunoreactivity in the right lesioned striatum **(A)**. After treatment (15 min) with the partial D1 dopamine agonist SKF38393 (2 mg/kg, i.p.), p-ERK1/2 is not evident in the dopamine-intact striatum **(B)** but is present in numerous neurons in the dopamine-depleted striatum **(C)**. To determine the type of striatal neuron in which p-ERK1/2 is present, sections were processed to display both p-ERK1/2 with a green fluorescent label and enkephalin mRNA with a red fluorescent label **(D”)**. Nearly all p-ERK1/2-immunoreactive neurons (blue arrows) are enkephalin negative. Only a small number enkephalin-positive neurons display p-ERK1/2 immunoreactivity (yellow arrow), whereas the vast majority are p-ERK1/2 negative (orange arrows). **(II)** Demonstration of distinct mechanisms of D1 dopamine receptor-mediated gene regulation in the dopamine (DA)-intact and -depleted striatum, using the full D1 agonist SKF81297 alone or combined with other drugs. **(A–D)** *In situ* hybridization histochemical localization of mRNA encoding c-fos 45 min after different drug combinations: **(A)** SKF81297 (0.5 mg/kg); **(B)** SKF81297 (2.0 mg/kg); **(C)** SKF81297 (2.0 mg/kg) combined with the muscarinic receptor antagonist scopolamine (5 mg/kg); or **(D)** SKF81297 (2.0 mg/kg) combined with the D2 dopamine receptor agonist (1 mg/kg) and scopolamine. The low dose of agonist alone **(A)** demonstrates the supersensitive response by the selective induction of c-fos in the dopamine depleted striatum. Bilateral induction of c-fos IEG in both the dopamine-intact and -depleted striatum follows treatment with high dose of the full D1 agonist alone **(B)** or in combination with other drugs **(C,D)**. However, when animals receiving any of these treatments (15 min survival) p-ERK1/2-immunoreactive neurons are evident only in the dopamine-depleted striatum and not in the dopamine-intact striatum (data not shown). The treatment combining full D1 agonist with both the D2 agonist and scopolamine produces the most robust c-fos IEG response in both the DA-intact **(E)** and DA-depleted **(F)** striatum at 45 min. This treatment also results in persistent p-ERK1/2 **(H)** and phosphorylated c-jun **(J)** in the dopamine-depleted striatum but does not activate p-ERK1/2 **(G)** or phosphorylated c-jun **(I)** in neurons in the dopamine-intact striatum. These results demonstrate that, although D1 dopamine receptor-mediated induction of the IEG c-fos occurs in both the dopamine intact and -depleted striatum, activation of ERK1/2 occurs only in the dopamine-depleted striatum (Gerfen et al., 2002).

In the rodent model of Parkinson’s disease depletion of striatal dopamine with unilateral lesions of the nigrostriatal dopamine pathway helped establish distinct functions of direct and indirect striatal pathways. A major finding using this model were effects of selective dopamine agonists on gene regulation following dopamine depletion that produced a supersensitive response. As many of the IEGs that are induced in response to dopamine agonists have been implicated in various forms of neuronal plasticity, a question was whether repeated enhanced activation of such genes underlie the development of dyskinesias that occurs following long-term treatment of patients with Parkinson’s disease. Induction of IEGs are mediated in dSPNs by coupling of the Drd1 and Drd2 receptors through Gs and Gi to increase or decrease cAMP levels, which activate protein kinases such as protein kinase A (PKA) that activate transcription factors to regulate gene expression of IEGs and others to modify neuronal plasticity and physiology (Sheng and Greenberg, 1990). In addition to PKA, the MAP kinase ERK1/2 was demonstrated to be activated by stimulation of corticostriatal excitatory inputs in SPNs (Sgambato et al., 1998). As activation of ERK1/2 was implicated in synaptic plasticity (Impey et al., 1999; Thomas and Huganir, 2004) activation of ERK1/2 determined by immunohistochemical labeling of phosphorylated ERK1/2 was examined in the unilateral dopamine lesion model following acute pharmacologic treatments (Gerfen et al., 2002). In response to partial Drd1 agonist treatments (SKF38393 2.0 mg/kg, i.p.) phospho-ERK1/2 immunoreactivity labeled the majority of dSPNs throughout the striatum in the dopamine depleted striatum whereas in the dopamine intact striatum labeled dSPNs were confined to the ventral striatum including the nucleus accumbens (Figure 3). To determine whether Drd1-mediated activation of ERK1/2 occurs only in the dopamine denervated striatum animals received treatments with high doses of a more potent Drd1 selective agonist (SKF81297) as well as with Drd1-agonists combined with Drd2 receptor- and muscarinic receptor agonists, which had been shown to induce IEGs in the dopamine intact striatum (Figure 4). The unilateral lesion model was particularly suited for this purpose to compare the effects of dopamine depletion. Using this paradigm, treatment with a relatively low dose of the Drd1-receptor agonist SKF81297 (0.5 mg/kg i.p.) produced little induction of c-fos in the dopamine intact striatum while producing very strong response in the dopamine denervated striatum, demonstrating the typical supersensitive response. Increasing the dose of Drd1 receptor agonist (2.0 mg/kg, i.p.) produced some c-fos induction in the intact striatum but less than on the lesioned side. Treatments combining the Drd1 agonist with a muscarinic receptor agonist (scopolamine) or with a Drd2 agonist (quinpirole) and scopolamine produced a c-fos response in the intact striatum that was comparable to the supersensitive response in the lesioned striatum. However, with each of these treatments, activation of ERK1/2, indicated by the labeling of dSPNs with phospho-ERK1/2 immunoreactivity, occurred in more than 85% of dSPNs throughout the striatum on the lesioned side but only in the ventral most striatum and nucleus accumbens in the intact striatum. These results suggested that following dopamine denervation of the striatum there is an alteration in coupling of the Drd1 receptor to signaling transduction mechanisms to activate ERK1/2 in dSPNs.

In the dopamine intact striatum activation of ERK1/2 occurs in the striatum in different conditions. Stimulation of the excitatory cortico-striatal pathway produced activation of ERK1/2 in the dopamine intact striatum primarily in iSPNs (Sgambato et al., 1998; Gerfen et al., 2002) through NMDA receptor coupling to Ca^2+^/calmodulin signaling systems that activate mitogen-activated protein kinase kinase (MEK) responsible for phosphorylation of ERK1/2 (Thomas and Huganir, 2004). Treatment with Drd2 receptor antagonists, including haloperidol and eticlopride, activated ERK1/2 throughout the striatum (Gerfen et al., 2002; Bertran-Gonzalez et al., 2008), possibly due to blockade of normal suppression of cortico-striatal stimulation of iSPNs by dopamine acting through Drd2 receptors. Activation of ERK1/2 has been implicated in psychostimulant addiction. A model proposed was that psychostimulants amplify NMDA-mediated ERK1/2 activation through D1 coupled to PKA, which through a mechanism involving dopamine- and cAMP-regulated phosphoprotein-32 (DARPP32) amplifies the activation of ERK1/2 (Greengard et al., 1999; Valjent et al., 2000, 2005). Psychostimulant activation of ERK1/2 was similar to that of Drd1 agonist treatment in the dopamine intact striatum in displaying regional variations with the most robust effects in the medial most and ventral striatum including the nucleus accumbens. Psychostimulants produced slightly increased numbers of SPNs in the dorsal striatum but only scattered neurons relative to the over 90% of dSPNs labeled in response to Drd1 in the dopamine depleted striatum (Gerfen et al., 2008). Of note was the finding that in mice with either a genetic deletion of Drd1 or DARPP-32, there was no difference in the number and distribution of phospho-ERK1/2 labeled SPNs in the dorsal and medial striatum compared to controls, while there was a significant reduction in the number of labeled neurons in the ventral striatum and nucleus accumbens (Gerfen et al., 2008). This suggests that psychostimulants activate multiple signal transduction pathways, one of which involves Drd1 receptor coupled though PKA and DARPP32 and another that is independent of dopamine signaling, likely involving the NMDA receptor.

**FIGURE 5:**
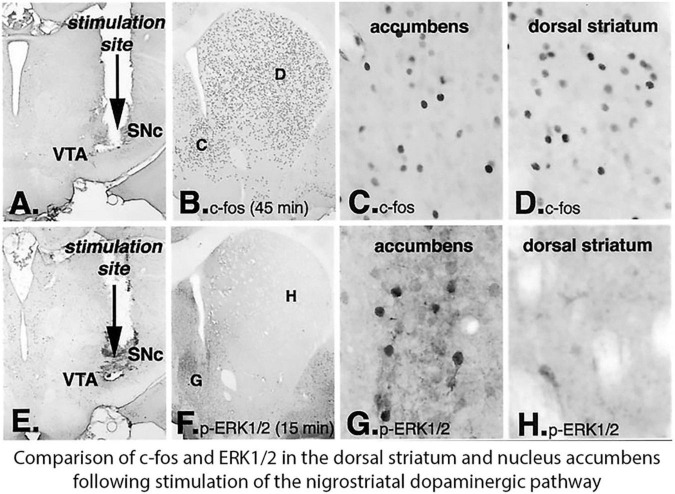
Electrical stimulation of the nigrostriatal pathway results in the induction of the IEG c-*fos* throughout the striatum and nucleus accumbens, but activation of ERK1/2 is restricted principally to the nucleus accumbent. Electrodes were placed in the junction between dopamine (DA) neurons in the ventral tegmental area (VTA) and substantia nigra pars compacta (SNc) and stimulated **(A,E)**. In animals killed 45 min after stimulation onset **(A–D)**, c-fos is induced throughout the dorsal striatum and nucleus accumbens **(B)**. Higher-power photomicrographs reveal c-fos-immunoreactive nuclei in the nucleus accumbens **(C)** and in the dorsal striatum **(D)**. In animals killed 15 min after stimulation onset **(E–H)**, the time point that is optimal for detecting phosphorylated ERK1/2, immunoreactive neurons are observed only in the nucleus accumbens **(F)**. Higher power photomicrographs reveal numerous rons in the nucleus accumbens **(G)**, whereas in the dorsal striatum, only scattered large immunoreactive neurons are observed **(H)** and not in SPNs (Gerfen et al., 2002).

Comparison of the induction of IEGs and activation of ERK1/2 in the striatum following electrical stimulation of the nigrostriatal dopamine pathway revealed regional differences in the dopamine intact striatum with the dopamine depleted striatum (Gerfen et al., 2002). Stimulation of the nigrostriatal pathway with electrodes placed in the substantia nigra pars compacta resulted in induction of the IEG c-fos throughout the striatum (Figures 5A–D), while producing phosphorylation of ERK1/2 that was restricted to dSPNs in the nucleus accumbens and only scattered weakly labeled SPNs in the dorsal striatum with some large aspiny neurons (putative cholinergic striatal interneurons) labeled as well (Figures 5E–H). This pattern was similar to Drd1 agonist activation of ERK1/2 in the dopamine intact striatum (Figure 4B) but contrasted with the labeling of the majority of dSPNs in the dorsal striatum of the lesioned striatum (Figure 4C). Taken together these studies suggested that following dopamine denervation there is a change in signal transduction mechanisms mediating D1 receptor activation of ERK1/2 compared to the intact striatum.

Clinical treatment of Parkinson’s disease with L-DOPA had long been the standard treatment that reversed bradykinesia, but with long term treatment invariably led to the development of dyskinesias, which limited its effectiveness (Bergmann et al., 1987; Nutt, 1990). Based on the finding that dopamine denervation resulted in a switch in Drd1-mediated activation of ERK1/2 in dSPNs it was proposed that this activation might produce an aberrant change in synaptic plasticity that might be responsible for LIDs produced after repeated treatment (Gerfen et al., 2002). Using a rodent model numerous studies have demonstrated a critical role of ERK1/2 in LID (Pavón et al., 2006; Santini et al., 2007; Westin et al., 2007). In addition to showing a direct correlation between the severity of LIDs and activation of ERK1/2, inhibitors of mitogen-activated kinase ERK kinase (MEK) that phosphorylates ERK1/2 suppressed both ERK1/2 activation and LIDs (Santini et al., 2007). A number of different factors and cellular processes have been related to ERK1/2 activation and LIDs including deltaFosB (Westin et al., 2007), a tyrosine phosphatase associated with the D1 receptor Shp-2 (Fiorentini et al., 2013), the metabotropic glutamate receptor gluR5 (Fieblinger et al., 2014), and DARPP32 (Santini et al., 2007) and a variety of others linking D1 receptors to signaling systems that regulate gene expression.

In striatal SPNs activation of ERK1/2 occurs in direct response to glutamate binding NMDA receptors linked to Ras signaling pathways coupled through MEK to ERK1/2 (Svenningsson et al., 2004; Valjent et al., 2005; Pascoli et al., 2011; Cahill et al., 2014). Evidence for amplification of NMDA activation of ERK1/2 by D1 dopamine receptors came from the effects of psychostimulants, which implicated D1 receptor coupled to the stimulatory G protein, Golf to cAMP mediated PKA activation of the protein phosphatase cascade including DARPP32 suppression of a protein phosphatase (PP1) to amplify activation of MEK and ERK1/2 (Valjent et al., 2005; Bertran-Gonzalez et al., 2008). The amplification of NMDA-activation of ERK1/2 signaling by dopamine acting through the D1 receptor was proposed as a mechanism by which coincident activation of glutamatergic and D1 dopamine receptors would result in synaptic plasticity to strengthen activated corticostriatal inputs to dSPNs (Valjent et al., 2005). This signaling pathway was also suggested to be responsible for activation of ERK1/2 in response to L-DOPA (Santini et al., 2007) as in mice with the DARPP-32 gene deleted there was a decrease in L-DOPA induced ERK1/2 phosphorylation following repeated treatments that produced LIDS, demonstrated with western blots. However, several studies demonstrated differences between psychostimulant and L-DOPA activation of ERK Using the same DARPP-32 knockout mice in the dopamine depleted striatum, acute treatment with L-DOPA resulted in immunohistochemical labeling of phosphorylated ERK1/2 comparable to control animals (Gerfen et al., 2008). While acute L-DOPA treatment produced activation of ERK1/2 in the dopamine depleted striatum in dSPNs (Gerfen et al., 2008) comparable to the labeling of most dSPNs with D1r agonist treatment (Gerfen et al., 2002), long term treatment with L-DOPA that produced LIDs resulted in a decrease in ERK1/2 activation in dSPNs coincident with an increase in activation in striatal cholinergic neurons (Ding et al., 2011). Discrepancy between these studies might be due to different methods used, in some activation of ERK1/2 with immunohistochemical labeling of phosphorylation of ERK1/2 in dSPNs (Gerfen et al., 2008; Ding et al., 2011), while the reported decrease in ERK1/2 activation in DARPP32 knockout animals used the western blot method (Santini et al., 2007).

Signal transduction mechanisms through which dopamine affects changes in synaptic plasticity and other physiologic functions in dSPNs and iSPNs differ between these neuron subtypes (Bertran-Gonzalez et al., 2008) as well as in different regions of the striatum (Gerfen et al., 2002). Regional differences are revealed by activation of ERK1/2 by psychostimulants primarily in the ventral striatum including the nucleus accumbens (Valjent et al., 2005) and the complementary pattern in the dopamine denervated dorsal striatum in response to D1r selective agonists and L-DOPA (Gerfen et al., 2008). Dopamine is generally considered to modify the physiologic response to excitatory input through glutamatergic NMDA and AMPA receptors, with Ca^2+^ influx through NMDA receptors triggering signaling to activate ERK1/2 (Valjent et al., 2005). In response to psychostimulants, D1 linkage through PKA to DARPP32 to amplify ERK1/2 activation triggered by NMDA Ca^2+^ influx appears to occur in dSPNs in the ventral striatum and nucleus accumbens (Valjent et al., 2005; Gerfen et al., 2008). Treatment with D1 receptor agonists or L-DOPA also produces activation of ERK1/2 in the ventral striatum and nucleus accumbens, but in contrast does not in the majority of dSPNs in the dorsal striatum (Gerfen et al., 2002, 2008). Direct stimulation of the nigrostriatal dopamine pathway induces c-fos in the dorsal striatum but only activates ERK1/2 in the ventral striatum and nucleus accumbens (Figure 5) indicating that D1r mediated regulation of ERK1/2 differs in the dorsal and ventral striatum and that the signaling pathway responsible for induction of c-fos and other IEGs (Berke et al., 1998) is distinct from the ERK1/2 pathway.

**FIGURE 6:**
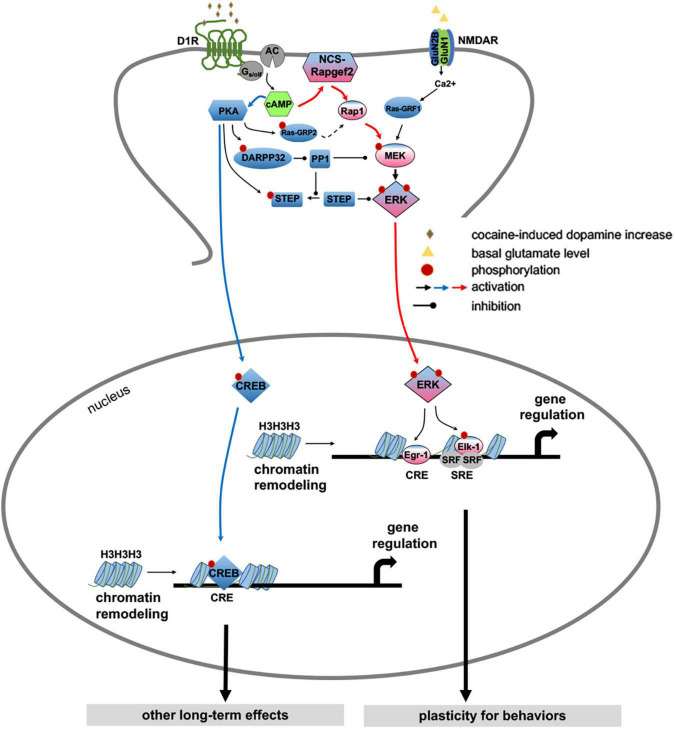
Proposed model for direct dopamine-dependent ERK1/2 activation in dSPNs (Jiang et al., 2021). Cocaine acts by increasing synaptic dopamine leading to ERK activation in NAc, required for locomotor sensitization and CPP. It has previously been proposed that D1 receptor activation affects ERK activity only indirectly, via PKA- and DARPP-32/STEP-mediated inhibition of ERK dephosphorylation (Svenningsson et al., 2004; Valjent et al., 2005), whereas direct ERK activation itself occurs in the D1 MSNs only via NMDAR-dependent glutamatergic signaling (Pascoli et al., 2011; Cahill et al., 2014) through calcium-sensitive Ras-guanine nucleotide releasing factor (Ras-GRF1) (Farnsworth et al., 1995; Fasano et al., 2009). We propose here a more parsimonious mechanism for ERK-dependent cocaine-induced dopaminergic signaling, in which cAMP elevation by dopamine in D1-MSNs results in parcelation of signaling between ERK and CREB with separate cellular consequences under the control of each pathway. An indirect modulatory role for PKA-dependent ERK phosphatase inhibition after psychomotor stimulant administration (Valjent et al., 2005; Sun et al., 2007), and additional ERK regulation by glutamatergic input to D1 dopaminoceptive neurons in the context of cellular plasticity underlying cocaine addiction (Valjent et al., 2000; Park et al., 2013; Cahill et al., 2014; Pascoli et al., 2014) is not contradicted by this model. We posit that D1 receptor activation, and cAMP elevation, in D1 MSNs likely results in parallel effects on ERK, both directly via NCS-Rapgef2 and indirectly via PKA with the effects of cocaine requiring multiple necessary, but perhaps individually insufficient inputs activated by dopamine, that converge on D1-MSN ERK phosphorylation. These include PKA/DARPP/STEP/PP1 (Svenningsson et al., 2004), PKA/RasGRP2/Rap1 (Nagai et al., 2016a,b), a NMDAR-dependent Ras activation (Fasano et al., 2009; Pascoli et al., 2011), and NCS-Rapgef2/Rap1/B-Raf/MEK.

Linkages between GPCRs such as dopamine receptors and ion channels such as NMDA glutamatergic receptors with signaling pathways effecting cell functions provide multiple sites for regulation. Proposed models consider that Drd1 linkage to the signaling pathways occurs through PKA, which acts as a cAMP sensor to phosphorylate downstream phosphatases and other proteins, which regulates phosphorylation of kinases upstream of ERK1/2 that are directly activated by the influx of Ca^2+^ through NMDA receptors through calcium sensitive Ras-guanine nucleotide releasing factor (Ras-GRF1) (Valjent et al., 2005; Pascoli et al., 2011; Gutierrez-Arenas et al., 2014). In these models the D1r and NMDA pathways converge in the activation of ERK1/2 that regulates activation of transcription factors including CREB (cAMP response element-binding protein), Egr1 and Elk1 among others to effect gene expression. However, induction of c-fos and other IEGs in dSPNs that are induced by CREB occur in the absence of ERK1/2 activation (Gerfen et al., 2002, 2008), suggesting that gene regulation involving CREB and ERK1/2 occur through dissociable pathways. Recent studies identified a novel cAMP sensor, Rapgef2, expressed in dSPNs that provides a separate cAMP signal transduction pathway that is necessary for D1r mediated activation of ERK1/2 in dSPNs (Jiang et al., 2017, 2021). Both psychostimulant and Drd1 agonist treatment activation of ERK1/2 in the nucleus accumbens is blocked by deletion of Rapgef2 in nucleus accumbens dSPNs (Jiang et al., 2021). Cocaine treatment, either acutely or with schedules that produce locomotor sensitization or conditioned place preference, results in activation of ERK1/2 in dSPNs in the nucleus accumbens, which are blocked by deletion of Rapgef2 in the nucleus accumbens. Additionally, with these treatment paradigms, in the nucleus accumbens activation of ERK1/2 and induction of the IEGs c-fos are unaffected by deletion of Rapgef2. A similar result occurs in hippocampal dentate granule neurons, in which deletion of Rapgef2 eliminates activation of ERK1/2 but not CREB (Jiang et al., 2017). These studies identify multiple cAMP signaling pathways in dSPNs, providing mechanisms for different cellular functions to be regulated independently or jointly (Figure 6). Direct activation of the ERK1/2 pathway occurs through either glutamatergic NMDA Ca^2+^ influx (Pascoli et al., 2011) or Drd1 coupled to cAMP sensor Rapgef2 (Jiang et al., 2021) and indirectly through Drd1 coupled through cAMP sensor PKA-dependent inhibition of ERK1/2 phosphatases (Valjent et al., 2005). Activation of ERK1/2 is responsible for regulation of many genes due to its phosphorylation of various transcription factors underlying changes in synaptic plasticity and other adaptations. On the other hand, while PKA does directly phosphorylate some transcription factors, including CREB, to regulate expression of some genes, there are many other target substrates that directly and more immediately affect neuronal excitability, protein trafficking and synaptic plasticity. From studies of the multiple mechanisms by which cellular function in the striatum is regulated a general simple principle that emerges is that dSPNs and iSPNs have both common and distinct properties that are regionally variant. A significant regional difference is evident by the limitation of coupling of the Drd1 receptor to activation of ERK1/2 in response to psychostimulants and Drd1 agonists to dSPNs in the nucleus accumbens and ventral striatum, while following dopamine denervation of the striatum results in switch to D1r coupled ERK1/2 activation in most dSPNs in the dorsal striatum.

## Physiologic and morphologic properties of dSPNs and iSPNs

A major area of research is to determine the functional cellular effects of dopamine mediated activation of signal transduction pathways (Kreitzer and Malenka, 2008; Pascoli et al., 2014). An example is the demonstration that repeated psychostimulant treatment increases both dendritic branching and spine density in dSPNs that is dependent on activation of ERK1/2 (Ren et al., 2010). Dendritic morphology has been shown to directly affect the physiology of SPNs demonstrating common and different properties of dSPNs and iSPNs in both the intact and dopamine denervated striatum. The resting potential of both types is maintained by the domination of inwardly rectifying Kir2 K+ channels in a down state at a hyperpolarized level far below the threshold to generate spiking activity (Wilson, 1993; Shen et al., 2007). In response to excitatory glutamatergic input from the cortex, which is coordinated both spatially and temporally, Kir2 K+ channels are overwhelmed resulting in depolarization of SPNs close to the spike threshold (Wilson and Kawaguchi, 1996; Day et al., 2008). When in this “up-state” SPNs spike in response to excitatory cortical inputs that are responsible for activity in the SPN output pathways. Interestingly cortical inputs responsible for spiking activity are not correlated with patterns of input responsible for the transition from the down to up-state (Stern et al., 1998). Consistent with the opposite effects of dopamine on gene regulation in SPNs (Gerfen et al., 1990), in iSPNs D2 receptor signaling in iSPNs diminishes both up-state transitions and spiking activity while D1 receptors have the opposite effect in dSPNs (Surmeier et al., 2007). Although there is a common morphology of SPNs, the number of dendrites and total dendritic length of dSPNs is significantly greater than those of iSPNs, which accounts for dSPNs receiving 50% more glutamatergic synapses than iSPNs (Gertler et al., 2008). These morphologic differences account for iSPNs being significantly more excitable than dSPNs (Gertler et al., 2008).

Changes in dendritic morphology and physiologic properties of dSPNs and iSPNs were studied following striatal dopamine denervation with treatments with L-DOPA that produced dyskinesias by Surmeier, Cenci and their colleagues (Fieblinger et al., 2014). Dopamine denervation resulted in a significant decrease in both dSPN and iSPN of dendritic branching and length, which were unaltered by L-DOPA treatment. As reported earlier (Day et al., 2006), there was a significant decrease in spine density of iSPNs, which was reversed by high dose L-DOPA treatment that produced dyskinesia. In dSPNs, dopamine denervation did not affect spine density, though both low and high dose L-DOPA resulted in a significant decrease in spine density and due to the decrease in dendrite branching and length a further reduction in spine number. Consistent with their physiologic properties in controls (Surmeier et al., 2007), the loss of dopamine in the striatum respectively increased and decreased excitability in dSPNs and iSPNs. These changes in the current required to generate action potentials are attributed to homeostatic mechanisms reflecting increased activity in iSPNs relative to dSPNs in the dopamine denervated PD model (Fieblinger et al., 2014). L-DOPA treatment normalized excitability in iSPNs and partially reversed the changes in dSPNs. Optogenetic and electrophysiologic methods demonstrated that cortico-striatal connectivity to dendritic spines was reduced in both dSPNs and iSPNs following dopamine denervation. High dose L-DOPA treatment that produced dyskinesia reversed the decrease in cortico-striatal spinous connectivity in iSPNs while producing a further reduction in dSPNs. This study demonstrated changes in dendritic morphology and the physiology of dSPN and iSPNs resulting from striatal dopamine denervation and L-DOPA treatment, with the only one exclusively associated with LID was the restoration of axospinous synapses (Fieblinger et al., 2014). While this points to the involvement of iSPNs in the development of LIDs, adaptations of the number and strength of cortico-striatal axospinous connections in both SPN types suggest possible reorganization and pruning of circuits involving specific patterns of connectivity. An example is provided by using the FosTRAP (targeted recombination in active populations) technique to express Cre-recombinase in neurons active during LID (Girasole et al., 2018). Striatal neurons labeled with FosTRAP during LID were a subset of dSPNs. When TRAPped neurons were activated optogenetically dyskinesias were produced without L-DOPA treatment, while selective suppression of these neurons ameliorated LIDs in response to L-DOPA.

## Normal function of the direct and indirect striatal projection pathways

Original concepts of the role of the basal ganglia in the generation of voluntary movements focused on activity from the cerebral cortex through the direct striatal pathway inhibiting the output of the GPi and SNr to disinhibit thalamic and brainstem circuits that generate movements (Chevalier et al., 1985; Deniau and Chevalier, 1985). The idea of imbalanced activity in the direct and indirect pathways as responsible for bradykinetic and dyskinetic clinical disorders introduced the concept that while the direct pathway promoted movement the indirect pathway suppressed movement with increased excitatory input from the STN to the SNr and GPi (Crossman, 1987; Albin et al., 1989; DeLong, 1990). Generation of eye movement saccades during pauses in SNr activity coincident with disinhibition of superior colliculus neurons responsible for saccades provided an example of activity in the direct pathway promoting movement (Hikosaka and Wurtz, 1983). Activity in the indirect pathway during an eye movement task that required a saccade to fixate on a target was revealed with increased activity in different STN neurons during different phases of the task, some increasing activity prior to and remaining active following a saccade to the contralateral field (Matsumura et al., 1992). This increase in STN activity was interpreted as suppressing unwanted saccades during fixation through increased inhibition of the SC by the SNr (Matsumura et al., 1992). Similar findings were obtained with studies of the activity of GPi neurons during limb movements in primates, which as with SNr neurons provide tonic inhibitory inputs to the thalamus. Of limb movement related neurons, 70% show increased activity and 30% decreased activity (Anderson and Horak, 1985; Brotchie et al., 1991; Mink and Thach, 1991) with corresponding mixed increased and decreased activity of thalamic neurons targeted by the GPi (Anderson et al., 1993). From such studies came the insight that actions involve both promoting and inhibiting distinct elements of movement, which led to the model of the basal ganglia that activity of direct and indirect pathways is coordinated to select particular motor pattern generators (MPGs) and to inhibit competing MPGs (Mink, 1996). Examples of the opponent effects of activity through these pathways include turning off motor programs to suppress antagonist muscles involved in wrist flexion to allow flexion of agonist muscles and suppression of muscles contracted to maintain a resting posture to allow execution of reaching movements to a target (Mink and Thach, 1993). Lesion studies and recordings of GPi units during more complex behaviors, including performance of movement sequences revealed complex patterns of increased and decreased activity in different neurons related to preparatory and movement phases of movement sequences (Brotchie et al., 1991; Strick et al., 1993). These studies were consistent with indirect pathway activity suppressing prior MPGs in anticipation of activation of new MPGs through the direct pathway (Mink, 1996).

## Molecular genetic research tools to study functional neuroanatomy

Innovative technical advances developed in the 2000s revolutionized functional neuroanatomical study of brain circuits (Luo et al., 2014). Prior to this, axonal tracing, neurophysiologic and genetic techniques had established many of the general principles of the functional organization of basal ganglia circuits but were limited by difficulties in studying functions of neuron subtypes, especially when they were intermingled as is the case of dSPNs and iSPNs. Experimentally manipulating circuits was possible with pharmacologic agents when there were receptors specifically expressed in a neuron subtype. Cell types could also be targeted by use of antisense mRNA genetic constructs or retrograde labeling of neurons based on unique axonal projection patterns. While genetic and sequencing techniques had improved through the 1990s only a fraction of those expressed in the brain had been identified. Sequencing of the human and mouse genome led to the generation of transgenic mouse lines expressing first GFP (Gong et al., 2003) and then Cre-recombinase (Gong et al., 2007; Gerfen et al., 2013) in a wide range genetically defined neuron subtypes. Combining use of these lines with viral vectors for neuroanatomical tracing (Wall et al., 2010; Oh et al., 2014) and innovative molecular genetic techniques to optogenetically activate and inhibit neurons (Boyden et al., 2005; Arenkiel et al., 2007; Petreanu et al., 2007) or to record Ca^2+^ influx as a measure of neuronal activity (Grienberger and Konnerth, 2012) opened the floodgates of studies of the function of neural circuits related to behavior.

As part of the NINDS/NIMH GENSAT project transgenic lines were generated to express Cre-recombinase in neuron subtypes in cortical and basal ganglia circuits, including neurons those that express Drd1 in dSPNs and Drd2 or Adora2 in iSPNs (Gong et al., 2007; Gerfen et al., 2013). In a study using these BAC-Cre transgenic mice and viral vectors providing Cre-dependent Channelrhodopsin-2 expression, optogenetic stimulation of Drd1-dSPN and Drd2-iSPN neurons respectively increased or suppressed motor activity (Kravitz et al., 2010). This finding directly demonstrated the opponent effects on behavior of the direct and indirect pathways when they are activated independently. During the normal execution of behavior Mink (1996) had proposed that actions comprise both activation and suppression of movement elements, which predicted concurrent activity in both dSPNs and iSPNs. That both dSPNs and iSPNs are active during the initiation of movement was demonstrated using viral vectors to express GCaMP in Drd1- or Drd2-Cre expressing striatal neurons to measure changes in neuronal activity with probes inserted into the striatum (Cui et al., 2013). Other studies using a GRIN lens inserted into the striatum, which allowed GCaMP imaging of the spatial distribution of active neurons, demonstrated that during normal motor activity separate clusters of dSPN and iSPN neurons display coordinated activity (Barbera et al., 2016; Parker et al., 2018). These findings were predicted by the model by Mink (1996) that activity in both pathways contribute to ongoing movement behavior.

That the direct pathway promotes selected actions while the indirect pathway suppresses competing motor programs does not adequately convey the complexity of the motor behaviors studied, primarily in primates, used to formulate the model of the opponent roles of these pathways (Mink, 1996). Using molecular genetic techniques to analyze and manipulate activity of SPNs in mice performing tasks comparable to those routinely used in primates Costa and his colleagues have begun to unravel differences in the activity in the direct and indirect pathway during complex behaviors (Cui et al., 2013, Jin et al., 2014; Tecuapetla et al., 2016). Study of tasks that require a series of distinct movements are instructive in being able to decompose specific elements of actions. Using a behavioral task that required a mouse to press a lever four times with increasing speed, four changes in activity in the direct and indirect pathways related to learning and performance of the task were analyzed (Jin et al., 2014). As task learning progressed, several distinct activity patterns were identified that encoded the entire sequence rather than individual lever presses. Some displayed sustained activity during the duration of the sequence performance (sequence related units), with others active at either the initiation or termination of the sequence (start/stop units). Both dSPNs and iSPNs displayed start/stop activity, while dSPNs displayed sustained sequence related activity and iSPNs displayed sequence related inhibition. These results showed that there is concomitant activity in both pathways to initiate the sequence, but their activity is different during execution and performance. Using a similar task, the specific contributions of dSPNs and iSPNs to initiation and execution of a sequence task were studied (Tecuapetla et al., 2016). In this study, using a sequence lever pressing paradigm that provided a multistep initiation process and lever pressing schedule that provided a measure of performance, activity of dSPNs and iSPNs were either inhibited or stimulated optogenetically during different phases of the task. Inhibition of either pathway increased the latency to initiate the action sequence, but for different reasons. Inhibition of dSPNs slowed the initiation of the sequence task, but did not affect performance, whereas inhibition of iSPNs resulted in the animal aborting the task and switching to another behavior. When optogenetic inhibition was delivered to either the dSPN or iSPN type after the first lever press, the number of lever presses was decreased but again for different reasons, only inhibition of iSPNs resulted in the animals aborting the sequence task. Similar differences were found with optogenetic activation after the first lever press, stimulation of dSPNs resulted in increased continued lever pressing, whereas after stimulation of iSPNs animals aborted the sequence task. Different effects that stimulation of dSPNs and iSPNs during different times of a complex sequence task demonstrated that action sequences are organized in a hierarchical manner, with the two pathways having distinct functions (Geddes et al., 2018). Many studies of the role of the striatal pathways use behaviors in which animals are trained to perform a particular sequence of actions for a reward. Using a novel approach analysis of dSPNs and iSPNs activity while animals engaged in spontaneous open field behavior (Markowitz et al., 2018). While moving around in an open area, the movement of an outline of a mouse’s spine was digitized and application of an unsupervised machine learning algorithm identified sub-second behavioral motifs. These were termed “syllables,” including rearing, diving, scrunching and locomoting, which animals performed spontaneously in different sequences. Analysis of activity in dSPNs and iSPNs was found to be correlated with different the behavioral syllables. When activity in the pathways was recorded simultaneously examples showed opponent activity in dSPNs and iSPNs during locomotion syllables, while during execution of other syllables only one pathway displayed changes in activity. Taken together these studies provide details of the opponent functions of the direct and indirect pathways in volitional behavior (Mink, 1996), by demonstrating that the pathways provide coordinated and complementary functions with the direct pathway involved in the initiation and performance of selected actions while the indirect pathway provides a permissive function by inhibiting competing actions or promoting switching behavior when appropriate.

## Indirect pathway circuits through the GPe

**FIGURE 7:**
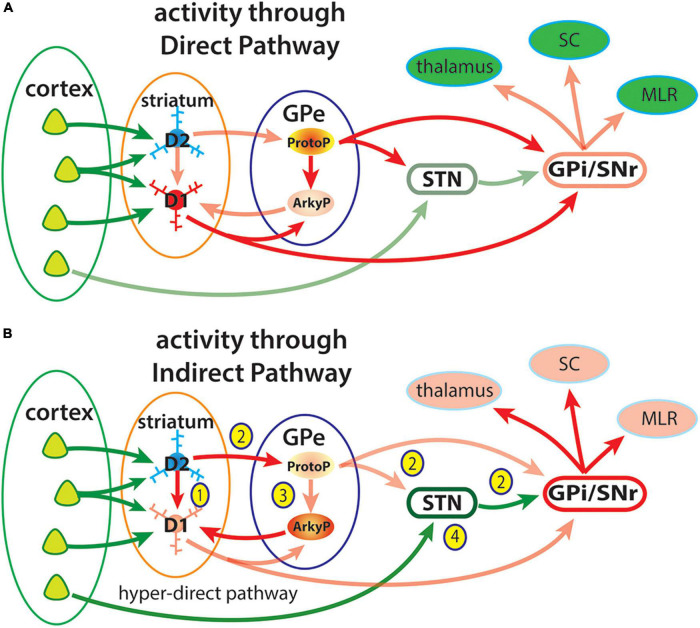
Diagram of simplified view of activity through the direct and indirect pathways highlighting some connections of the GPe. Cortical neurons provide excitatory inputs to D1r- or D2r-expressing SPNs that give rise to the direct and indirect striatal pathways to the output pathways of the basal arising from GABAergic neurons in the GPi and SNr. These output neurons are tonically active, providing inhibitory input to circuits engaged in movement including the thalamus, superior colliculus (SC) and midbrain locomotor region (MLS). **(A)** Activity through the direct pathway: Cortical excitatory input to dSPNs (D1) provides direct inhibition of GPi/SNr neurons to disinhibit their targets in the thalamus, superior colliculus (SC) and midbrain locomotor region (MLR) (Chevalier et al., 1985). Prototypical GPe neurons are tonically active and provide inhibitory inputs to the subthalamic nucleus (STN) and GPi/SNr, which also contribute to disinhibition of basal ganglia targets. **(B)** Activity in the indirect pathway may have opponent effects on the direct pathway at multiple sites. (1) iSPNs may directly inhibit activity of neighboring dSPNs through collateral axons (Czubayko and Plenz, 2002; Taverna et al., 2008; Matamales et al., 2020). (2) The original indirect pathway circuit involves activity of iSPNs provide inhibition of prototypical GPe (ProtoP) neurons, which are tonically active, resulting in disinhibition of the STN and increased inhibition of basal ganglia targets (Pamukcu et al., 2020). (3) iSPN inhibition of ProtoGPe neurons also disinhibits Arkypallidal (ArkyP) neurons, which provide inhibitory inputs selectively to dSPNs resulting in suppression of movement (Aristieta et al., 2021; Cui et al., 2021a,b). (4) The hyper-direct pathway from the cerebral cortex directly to the STN provides an additional mechanism to increase the inhibitory output of the basal ganglia.

Early models of the indirect striatal pathway focused on the inhibitory projections of GPe neurons to the STN, whose glutamatergic neurons provide excitatory input to the GPi/SNr. Selective suppression of action elements resulting from iSPN activity was thought to result from disinhibition of STN neurons to increase inhibitory output to the thalamus and other targets, which formed the basis of successful treatments for Parkinson’s disease (DeLong, 1990; Benabid, 2003). However, recent studies of the GPe have revealed additional complexity of the role of the indirect pathway in suppressing actions that are attributed to connections of two main subtypes of GPe neurons. One subtype, termed the prototypical pallidal neuron (ProtoGPe) expresses the calcium binding protein Parvalbumin (PV), while the other, termed the other termed the arkypallidal (ArkyGPe), expresses the transcription factor Npas1 (Mastro et al., 2014; Pamukcu et al., 2020; Cui et al., 2021b). ProtoGPe neurons receive inputs from iSPNs and project to the STN and to GABAergic neurons in the GPi/SNr (Saunders et al., 2016), which is the circuit classically associated with motor suppression through the indirect pathway. Subtypes of ProtoGPe neurons have been described, some of which project selectively to the STN and GPi/SNr, while others project to the parafascicular and reticular thalamic nuclei (Mastro et al., 2014; Cui et al., 2021b; Lilascharoen et al., 2021). ArkyGPe neurons receive inputs from the striatum, selectively from dSPNs (Cui et al., 2021a) and project back to both dSPNs and iSPNs as well as to striatal interneurons (Mallet et al., 2012). Subtypes of ArkyGPe neurons have been identified that project to the SNc, reticular nucleus of the thalamus and to the STN, though to a distinct part distinct from that receiving inputs from ProtoGPe neurons (Cui et al., 2021b). A simplified diagram of these connections of the GPe in terms of how they contribute to activity in the direct and indirect pathways is provided in Figure 7.

A circuit within the GPe consists of excitatory STN inputs to ProtoGPe neurons, which through their local collaterals provides inhibition of ArkyGPe neurons (Aristieta et al., 2021; Ketzef and Silberberg, 2021). This circuit has recently been shown to provide an additional mechanism to suppress elements of movement (Aristieta et al., 2021; Dong et al., 2021; Ketzef and Silberberg, 2021). Inhibition of the STN or stimulation of ProtoGPe neurons increases locomotion (Pamukcu et al., 2020), consistent with the classic model of movement suppression by the indirect pathway. A novel mechanism of movement suppression involves direct inhibition of dSPNs by ArkyGPe neuron projections to the striatum (Pamukcu et al., 2020; Aristieta et al., 2021; Ketzef and Silberberg, 2021). When active, ProtoGPe neurons inhibit ArkyGPe neurons through a local axon collateral, such that iSPN inhibition of ProtoGPe neurons results in disinhibition of ArkyGPe neurons to inhibit of striatal dSPNs and suppress movements. This di-synaptic circuit in the GPe provides an additional mechanism of movement suppression, to that involving the indirect pathway through the STN to the SNr/GPi. Distinct functions have been ascribed to different components of GPe circuits. ProtoGPe neurons that project to the STN and GPi/SNr have been shown to affect locomotion, while those projecting to the thalamus are involved in reversal learning (Lilascharoen et al., 2021). ArkyGPe neurons have been proposed to provide a “stop” signal to terminate an ongoing movement (Mallet et al., 2016).

## Opponent functions of the direct and indirect pathways during actions

Connections of the direct and indirect pathways originating from dSPNs and iSPNs in the striatum through the ProtoGPe and ArkyGPe neurons (and others) of the GPe to the output nuclei of the basal ganglia are diagrammed in Figure 7. Advances in functional neuroanatomical techniques have revealed the remarkable specificity of connections between different neuron subtypes at each level through the pathway and how patterns of activity are generated in these circuits. Ascribing how such activity affects movements often considers activity of a single circuit component independent of activity in another. For example, the classic model that the direct and indirect pathways promote and suppress movement was based on imbalances in these pathways that produced clinical hyperkinetic and bradykinetic disorders (Albin et al., 1989; DeLong, 1990) and then substantiated by activation of each pathway independently (Kravitz et al., 2010). The insight that actions involve both facilitation of specific movements and suppression of others led to the proposal that activity in the direct pathway promotes selected motor programs while the indirect pathway suppresses competing or alternative motor programs (Mink, 1996). This model has been substantiated by studies demonstrating that both dSPNs and iSPNs are not only active during actions but active differentially during distinct phases of a sequence task (Cui et al., 2013, Jin et al., 2014; Tecuapetla et al., 2016; Markowitz et al., 2018).

To further study the opponent effects of activity in the direct and indirect pathway during normal behavior Chan and his colleagues (Cui et al., 2021a) used an innovative approach in which over 20 elements of an animal’s movement were measured during periods of locomotion, rearing, being motionless and performing fine movements. Measures of movement of different body parts, speed of movements, and frequency and duration of different behaviors were analyzed before and during optogenetic stimulation or inhibition of dSPNs and iSPNs in medial (DMS) and lateral (DLS) regions of the dorsal striatum. Hierarchical cluster analysis of the movement metrics revealed correlations between activation of different striatal neurons and coherent behavioral states, such as locomotion, rearing, remaining motionless and fine movements. Optogenetic stimulation of dSPNs and iSPNs within the same region produced opponent behaviors, such as promoting or suppressing locomotion. Most informative was the finding that the opponent behavioral effects varied between striatal regions. Stimulation of dSPNs and iSPNs in the DMS respectively promoted and suppressed locomotion, whereas the opposite was found when these neurons in the DLS were stimulated. Behaviors produced by stimulation of dSPNs and iSPNs in DMS and DLS were shown to often be complementary. During periods of increased locomotion correlated with activation of DMS dSPNs there was a suppression of motionless and fine movement behavior, whereas activation of DLS dSPNs were correlated with periods of fine movements and suppression of locomotion. A companion study dissected the function of GPe circuits using a similar experimental approach to correlate the activity of ProtoGPe and ArkyGPe neurons with different elements of motor behavior (Cui et al., 2021b). In this study the mouse lines expressing Cre in PV or Kcng4 subtypes of ProtoGPe neurons or Npas1 or Foxp2+ subtypes of ArkyGPe were used to allow either activation of inhibition of these neurons (these are referred to as ArkyGPe and ProtoGPe subtypes). Consistent with prior studies, activation of ProtoGPe and ArkyGPe respectively increase and suppress movement. When movement metrics including locomotion duration, speed, motionless duration and frequency were compared they showed opposite correlations with activation of ProtoGPe and ArkyGPe. Combining movement metrics to define behavioral states suggested that ProtoGPe and ArkyGPe neurons act cooperatively as opposing forces to tune behavior and regulate transitions between actions (Cui et al., 2021b).

These studies expand the conceptual model that the direct and indirect pathways function to promote selected actions and suppress alternatives (Mink, 1996). Rather than considering a single element such as locomotion as being promoted or suppressed, distinct elements of behaviors may be complexly correlated with activity in the direct and indirect pathways. In studies when well-trained sequence tasks are executed, activity in the direct and indirect pathways may promote or suppress like movement elements, which are perceived as providing “stop” and “go” signals. Studies of natural spontaneous behaviors reveal that activity in the direct pathway promoting a particular movement element may occur concurrently with activity in the indirect pathway suppressing a very different type of movement. Analysis of multiple metrics of movement provides a more comprehensive perspective of how actions are composed of multiple elements. An example is that the performance of fine movements occurs during motionless periods, which may be considered as suppression of locomotion (Cui et al., 2021a). Complex patterns of correlation and decorrelation of activity in dSPNs and iSPNs during performance of syllable components of spontaneous action sequences and support concepts that activity in the indirect pathway in some cases suppresses competing actions and in other cases cooperatively promotes selected actions (Markowitz et al., 2018; Cui et al., 2021b).

## Organization of corticostriatal inputs to the direct and indirect pathways

The effect of the direct and indirect basal ganglia pathways on behavior is determined by the organization of cortical input to the striatum. In the prevailing model of the organization of the basal ganglia, parallel circuits originate from functionally defined cortical regions that project through striatal projections to the output targets in the thalamus, which project back to the cortex (Alexander et al., 1986). Subregions of motor, sensory and association cortical areas are mapped based on cytoarchitectural, neuroanatomic, physiologic and behavioral analysis. Unlike the cortex, where there are distinct boundaries between functionally defined cortical areas, there are no clear boundaries within the striatum that distinguish such areas. The development of neuroanatomical techniques using viral vectors to label projections of specific neuron subtypes have provided a detailed and comprehensive mapping of functional zones through the levels of the basal ganglia circuits (Hintiryan et al., 2016; Lee et al., 2020; Foster et al., 2021). Functional zones within the striatum are determined by the cortical areas that they receive input from. On the one hand corticostriatal projections are organized in a general topographic manner, on the other hand, cortical areas project broadly to the striatum such that there is considerable overlap of inputs from different areas. The structure of the convergence of information from different cortical areas to individual SPNs within a striatal region determines its function. In general, cortical areas that are connected provide overlapping striatal inputs (Yeterian and van Hoesen, 1978; Hooks et al., 2018). Using a computational neuroanatomic approach that incorporated analysis of projections from over 150 axonal tracer injections into the cortex, 29 distinct striatal functional domains were defined based on the patterns of overlap from connected cortical areas (Hintiryan et al., 2016). In a subsequent study tracer injected into the 29 striatal functional domains revealed 14 functional areas within the SNr and 36 within the GPe (Foster et al., 2021). This study demonstrated that connections arising from cortical networks that define functional pathways to the striatum are maintained into the organization of the direct and indirect pathways. Interestingly their data suggest that there is more convergence of projections in the direct pathway to the SNr from different striatal zones while projections to the GPe remain more precisely segregated. Distinct functional effects on motor behavior of striatal zones identified based on patterns of cortical input was demonstrated by the generation of different direction of licking or turning when dSPNs in different striatal zones were stimulated (Lee et al., 2020). The parallel topographic organization of direct pathway projections are maintained through the projections of the SNr its targets in the superior colliculus (SC) and in the ventral medial and parafascicular thalamus (Lee et al., 2020). The opponent effects of the direct and indirect pathways through the circuits originating in the striatum to the targets of the output nuclei of basal ganglia wasillustrated by a study that demonstrated that activity of iSPNs modulated activity in the superior colliculus that determined which of two actions is performed (Lee and Sabatini, 2021). Using a simple task in which an animal licks either right or left, iSPN activation suppressed contraversive licking and promoted ipsiversive licking. Activity in the ipsilateral SC was suppressed by iSPN activation, while activity in the contralateral SC was increased due to disinhibition through projections from the ipsilateral to contralateral SC. This study showed that within the targets of basal ganglia outputs there are circuits that have opponent effects on action selection.

Activity in the direct and indirect pathways is initiated by excitatory inputs from the cortex and thalamus. A critical question is whether individual cortical and thalamic neurons that provide inputs selectively target dSPNs and iSPNs or provide inputs to both. To address this question modified rabies virus was used to label cortical neurons that provide inputs to dSPNs or iSPNs that express Cre (Wall et al., 2013). Results demonstrated differences in the relative number of cortical neurons providing inputs to dSPNs and iSPNs varied dependent on the cortical area of origin. A larger number of corticostriatal neurons in motor and sensory areas were shown to provide inputs to iSPNs, whereas limbic cortical areas provided relatively more inputs to dSPNs. While these results did not demonstrate a complete segregation of inputs to dSPNs and iSPNs from any particular cortical area, they did suggest that there is some preferential difference. Also, this study did not determine if individual cortical neurons provide inputs to both SPN subtypes. Similar questions for thalamic inputs to dSPNs and iSPNs remain to be resolved.

### Summary

A core feature of the basal ganglia are the opponent functions of parallel pathways that process cortical input through the direct and indirect pathways to affect action selection. These pathways originate from dSPNs and iSPNs in the striatum. Opponent effects of dopamine on these neurons is a consequence of its stimulation of dSPNs through the Drd1 receptor and inhibition of iSPNs through the Drd2 receptor (Gerfen et al., 1990; Surmeier et al., 1996). Dopamine acting on Drd1 and Drd2 receptors couple through multiple signal transduction pathways to produce distinct physiologic responses in dSPNs and iSPNs, which affect their activity in response to excitatory input from the cortex and thalamus. Imbalance between the activity of dSPNs and iSPNs in hyperkinetic and bradykinetic clinical disorders was the basis of the model that the direct pathway promoted and the indirect pathway suppressed movement (Albin et al., 1989; DeLong, 1990). In clinical disorders one pathway’s effects may dominate over the other resulting in the inability to either initiate movements when the indirect pathway dominates or control excessive movement when the direct pathway dominates. The insight that normal behavior requires coordinated promotion of some movement elements and suppression of others was the basis of a proposal that activity in the direct pathway promotes selected actions while activity in the indirect pathway suppresses competing or alternative actions (Mink, 1996). Consistent with this model numerous studies demonstrated concurrent activity in dSPNs and iSPNs during movement and correlated respectively with promotion or suppression of different elements of actions in a variety of behaviors (Cui et al., 2013; Jin et al., 2014; Barbera et al., 2016; Tecuapetla et al., 2016; Markowitz et al., 2018; Parker et al., 2018; Cruz et al., 2022). Original concepts of the functions of the direct and indirect pathway focused on activity of dSPNs providing direct inhibition of GABAergic neurons in the GPi/SNr to disinhibit target circuits of the basal ganglia to promote selected actions, while activity of iSPNs inhibited GPe neurons to disinhibit STN neurons, thereby indirectly providing inhibition of the GPi/SNr to suppress actions. Studies have revealed circuits of the GPe, including connections of ProtoGPe and ArkyGPe neuron subtypes, which provide additional mechanisms within the indirect pathway to suppress actions. In the main, ProtoGPe neurons constitute the original indirect pathway connections with the STN and also provide collaterals to ArkyGPe neurons, which provide inhibitory projections back to dSPNs in the striatum. Activity of iSPNs that inhibits ProtoGPe neurons results in disinhibition of ArkyGPe neurons, which directly inhibit dSPNs and suppress actions, thus providing an additional mechanism for activity in the indirect pathway to suppress actions (Pamukcu et al., 2020; Aristieta et al., 2021; Ketzef and Silberberg, 2021). Studies of the effects of the direct and indirect pathways on natural spontaneous activity analyzing multiple measures of action elements expanded concepts of their functions beyond simply providing “go” and “no go” signals that adjust the vigor and speed of actions. Rather, a model in which activity in the pathways complement each other, with the direct pathway promoting the initiation and performance of selected actions while the indirect pathway functions in a permissive manner, allowing selected actions to occur, and aborting prior actions to allow switching to alternative actions (Tecuapetla et al., 2016; Markowitz et al., 2018; Cui et al., 2021a,b; Lee and Sabatini, 2021). The cerebral cortex encodes the wide range of behavioral options available to an animal to engage that are processed through the direct and indirect pathways (Cisek and Kalaska, 2010). Opponent mechanisms at multiple levels in these pathways, in the dSPNs and iSPNs in the striatum, in the ProtoGPe and ArkyGPe neurons in the GPe, and in the output targets of the GPi/SNr in the thalamus and midbrain motor centers, act cooperatively to generate coherent behavior. A challenge remains to determine how activity in parallel pathways originating in the cortex encode specific elements of actions in populations of dSPNs and iSPNs and how the opponent mechanisms at multiple levels through the direct and indirect pathways are integrated to generate coherent behavior.

## Author contributions

The author confirms being the sole contributor of this work and has approved it for publication.
